# Clinical significance of MATN1-AS1 as ceRNA of Mir-200b in tissues and serum of patients with cervical cancer

**DOI:** 10.1038/s41598-023-42023-2

**Published:** 2023-09-15

**Authors:** Lijie He, Pu Li, Heping Zhang

**Affiliations:** 1 Tianjin Fifth Central Hospital, Tianjin, 300450 China; 2 Department of Clinical Laboratory, Tianjin Fifth Central Hospital, 41 Zhejiang Road, Tianjin, 300450 People’s Republic of China; 3 Department of Gynaecology and Obstetricsclinical, Tianjin Central Obstetrics and Gynecology Hospital, Tianjin, 300400 People’s Republic of China

**Keywords:** Biochemistry, Cancer

## Abstract

To analyze the clinical significance of MATN1-AS1 as ceRNA of Mir-200b in the tissues and serum of cervical cancer patients. A total of 50 patients with cervical cancer admitted to our hospital from March 2018 to March 2019 were selected as the research objects. All patients underwent surgical resection of cancer tissues in our hospital, and cervical cancer tissues and adjacent tissues more than 2 cm away from the edge of cancer tissues were retained. Patients with cervical cancer were selected as the research group, and 50 patients with benign uterine lesions were selected as the control group. The expressions of MATN1-AS1 and Mir-200b in cervical cancer tissues and serum were detected by real-time PCR, and the correlation between MATN1-AS1 and Mir-200b was analyzed. The relationship between MATN1-AS1, Mir-200b and clinical features was analyzed, and the 3-year survival rate of cervical cancer patients was analyzed. Compared with adjacent tissues, the relative expression levels of MATN1-AS1 and Mir-200b in cancer tissues were significantly increased (*P* < 0.05). Compared with the control group, the relative expression levels of MATN1-AS1 and mir-200b in the study group were increased (*P* < 0.05). The expression levels of matn1-as1 and mir-200b were higher in poorly differentiated, tumor ≥ 4 cm, FIGO stage iii–iv, and lymph node metastasis patients (*P* < 0.05). Correlation analysis showed that MATN1-AS1 was positively correlated with Mir-200b (*r* = 0.625, *P* = 0.001). Compared with blank control group, the relative expression levels of MATN1-AS1 and Mir-200b in MATN1-AS1 silencing group were decreased (*P* < 0.05). The 3-year survival rate of 48 patients with cervical cancer was 66.67% (32/48). The survival rate of patients with high expression of MATN1-AS1 was lower than that of patients with low expression of MATN1-AS1, and the survival rate of patients with high expression of Mir-200b was lower than that of patients with low expression of Mir-200b (*x*^2^ = 4.251, 5.244, *P* = 0.011, 0.008). There is a potential binding point between MATN1-AS1 and Mir-200b. The expressions of MATN1-AS1 and Mir-200b are increased in the tissues and serum of cervical cancer patients, and they are positively correlated. Silencing of MATN1-AS1 in cervical cancer cell lines can reduce the expression of Mir-200b. Matn1-as1 can regulate the expression of Mir-200b and participate in the occurrence and development of cervical cancer.

## Introduction

Cervical cancer is common in females with high clinical incidence rate and mortality, mainly caused by HPV infection and the occurrence of cervical cancer is related to immunity, genetics and environment^1,2^. Surgical, radiotherapy and chemotherapy methods for cervical cancer has made great progress with the development of medical technology, but the prognosis of cervical cancer patients is poor. It is important to find new biomarkers and targets in the diagnosis and treatment of cervical cancer^3^. MATN1 antisense RNA 1(MATN1‐AS1) is a newly discovered Long non‐coding RNAs (lncRNAs) which is a non-coding RNA with a length of more than 200nt. It can participate in the regulation of genes and plays an important role in various physiological processes of the body and can be used as a structural component to regulate the stability and failure of mRNA^4^. miRNA is a non-coding single stranded RNA molecule which can inhibit the degradation or translation of target gene of mRNA and regulate target genes. miR-200b plays an important role in the development of cervical cancer. Alteration of variation sites in precursor miRNA sequences can affect the splicing and maturation process of miRNA, inhibit the expression of mature miRNA, and inhibit the combination of target gene and miRNA that miRNA gene variation plays an important role in the occurrence and development of tumors too^5^. Competing endogenous RNAs (ceRNAs) can combine with miRNA through miRNA reaction elements, inhibit miRNA function, and express the miRNA in vitro^6^.

Mir-200b overexpression had a pronounced radiosensitizing effect in cervical cancer tumor xenografts, Mir-200b downregulation is a candidate biomarker of cervical cancer central pelvic recurrence and seems to predict cell adhesion‐mediated tumor radioresistance independent of clinical markers and hypoxia.

In this study, we first discover that MATN1‐AS1 may play a significant role in cervical cancer after analysing TCGA database. Then, potential miRNAs that might have interactions with MATN1‐AS1 are screened using online tools starBase 2.0 from miR‐200 family is found out. Functionally, MATN1‐AS1 knockdown restrained cell proliferation but stimulated apoptosis in vitro and repressed tumour growth in vivo. Mechanistic investigations validated that MATN1‐AS1 functioned as a ceRNA for miR‐200b to upregulate FoxG1 which was also verified to exert a growth‐promoting role in cervical cancer cells here. Based on this, we aim to investigate the role and function of MATN1‐AS1 in cervical cancer and identify whether it affects cervical cancer by functioning as ceRNA for miR‐200b.

The paper analyzes the expression of MATN1-AS1 as miR-200b ceRNA in tissues and serum of cervical cancer patients in clinical significance analysis is based on there is no clinical study showing that MATN1-AS1 and miR-200b have a regulatory relationship.

## Materials and methods

### Participants

50 cases of cervical cancer patients admitted to our hospital from March 2018 to March 2019 were selected as the study subject investigated. All patients underwent surgical resection of cancer tissue in our hospital and reserved cervical cancer tissue and cancer adjacent tissue more than 2.0 cm from the edge of cancer tissue. Cervical cancer patients were selected as the study group with aged 42–55 (48.25 ± 3.17) years. In addition, 50 patients with benign uterine lesions were selected as the control group. All patients had been informed about the contents of the study and signed an informed consent form. This study was approved by the Ethics Committee of Tianjin Fifth Central Hospital.

#### Inclusion criteria

All patients met the diagnostic criteria for cervical cancer in the Expert Consensus on immunological Prevention of human Papillomavirus Related Diseases and were diagnosed pathologically^7^.

#### Exclusion criteria

The patients who received preoperative anti-tumor treatment, the patients with other malignant tumors, the patients with blood system diseases, the patients with an estimated survival period of ≤ 3 months and the patients suffered from thyroid disease.

### Chose fluorescence quantitative to detect the expression of MATN1-AS1 and miR-200b in tissues and serum

All subjects took 6 mL of fasting venous blood, centrifuged at a radius of 5 cm and with speed of 2000 r/min for 15 min, separated the upper serum, and stored it at – 80 °C for inspection. The cancer tissues and adjacent tissues of patients were taken to prepare tissue homogenization. MATN1-AS1 and miR-200b were detected by Real-time PCR, the Total RNA in tissues was extracted by Trizol, RNA in tissues was reverse transcribed into cDNA by Takara reverse transcription kit, Primer sequence was designed by Primer5.0 and the PCR instrument was started. Reaction system: Upstream and downstream primers(0.4 μL), cDNA template (1.0 μL), SYBGreen (10.0 μL), and added distilled water to 20.0 μL. Reaction conditions: A total of 40 cycles for pre-denaturation for 1 s, 5 s, 31 s under 95 °C and using 2^−ΔΔCt^ method to calculate the relative expression of MATN1-AS1 and miR-200b. MATN1-AS1 upstream primer sequence was 5′-CCGGCCTTGTTGTATACAGTCATTACTCGAGTAATGACTGTATACAACAAGGTTTTTTG-3′, downstream primer sequence was 5′-CCGGGCGCTCCTGTTTATGTACTTACTCGAGTAAGTACATAAACAGGAGCGCTTTTTTG-3′. miR-200b upstream primer sequence was 5′-UAAUACUGCCUGGUAAUGAUGA-3′, miR-200b downstream primer sequence was 5′-UUCUCCGAACGUGUCACGUTT-3′. Reference gene U6 upstream primer sequence was 5′-CATGAGAAGTATGACAACAGCCT-3′ and downstream primer sequence was 5′-AGTCCTTCCACGATACCAAAG-3′.

### Data collection and follow-up

The data of cervical cancer all patients (pathological type, degree of differentiation, tumor size, FIGO stage, lymph node metastasis) were statistically analysed and were followed up by outpatient with re-examination, telephone. A total of 2 patients were lost in the 3 years follow-up time.

### Cell transfection

Purchased human cervical cancer cell lines, laid a certain amount of cells on 6-well plates one day before transfection and put them into complete culture medium for culture. The cells were fused to 80–90% after 24 h. The cervical cancer cells were transfected with Lipofectamine 3000, the blank control group did not carry out any treatment, the silent MATN1-AS1 group transfected MATN1-AS1 silent plasmid (plasmid transfection concentration, 1 µg/L), and put it into 2% culture serum with a volume of 2 mL. The culture medium was changed after 5 h and cell RNA was extracted after 48 h to subsequent tests.

### Statistical analysis

The statistical analyses were performed using the Statistical Package for the Social Sciences version 19.0 (SPSS Inc., Chicago, IL, USA). The measurement data were expressed by mean ± standard deviation ($\overline{x} \pm s$) with t-test for comparison, homogeneity of variance test was used for comparison among groups. The correlation between the MATN1-AS1 and miR-200b was analyzed by Pearson correlation analysis. Kaplan Meier curve was used for survival analysis. P < 0.05 was considered as a statistically significant.

### Ethics approval and consent to participate

This study was approved by the Tianjin Fifth Central Hospital Ethics Committee, I confirmed that informed consent was obtained from all patients and their families, I confirmed all methods were carried out in accordance with Helsinki declaration.

## Results

### Relative expression of MATN1-AS1 and miR-200b in cervical cancer

The relative expression of MATN1-AS1 and miR-200b in cancer tissues was significantly higher adjacent tissues (P < 0.05) (Table 1).

**Table 1:** Relative expression of MATN1-AS1 and miR-200b in cervical cancer ($\overline{x} \pm s$).

Group	Case	MATN1-AS1	miR-200b
Cancer adjacent tissues	50	5.36 ± 0.74	0.49 ± 0.05
Cancer tissues	50	13.14 ± 2.35	1.24 ± 0.22
*t*		22.330	23.510
*P*		0.001	0.001

### Relative expression of MATN1-AS1 and miR-200b in cervical cancer serum

Compared with the control group, the relative expression of MATN1-AS1 and miR-200b in the study group significantly increased (P < 0.05) (Table 2).

**Table 2:** Relative expression of MATN1-AS1 and miR-200b in serum of cervical cancer ($\overline{x} \pm s$).

Group	Case	MATN1-AS1	miR-200b
Control group	50	4.85 ± 0.55	0.38 ± 0.04
Study group	50	12.05 ± 2.27	1.17 ± 0.20
*t*		21.800	27.390
*P*		0.001	0.001

### Relationship of expression of MATN1-AS1, miR-200b and clinicopathological characteristics in cancer tissues

The expression of MATN1-AS1 and miR-200b was not related to the pathological type, but the expression of MATN1-AS1 and miR-200b was related to the degree of differentiation, tumor size, FIGO stage and lymph node metastasis. The expression of MATN1-AS1 and miR-200b was higher in patients with poor differentiation, tumor size ≥ 4 cm, FIGO stage III–IV, and there was lymph node metastasis (*P* < 0.05) (Table 3).

**Table 3:** Relationship of expression of MATN1-AS1, miR-200b and clinicopathological characteristics in cancer tissues ($\overline{x} \pm s$).

Items	Case	MATN1-AS1	*F/t/P*	miR-200b	*F/t/P*
Pathological type					
Squamous cell carcinoma	24	13.16 ± 2.37	0.060/0.952	1.22 ± 0.20	0.637/0.527
Adenocarcinoma	26	13.12 ± 2.32		1.26 ± 0.24	
Degree of tumor differentiation					
Poorly differentiation	17	17.24 ± 2.75	4.890/0.001	2.41 ± 0.35	13.810/0.001
Moderate differentiation	21	13.17 ± 2.38		1.18 ± 0.19	
Well differentiation	12	8.14 ± 0.92		0.72 ± 0.08	
Tumor size					
< 4 cm	28	7.52 ± 0.83	18.480/0.001	0.85 ± 0.09	13.300/0.001
≥ 4 cm	22	18.27 ± 2.94		1.55 ± 0.26	
FIGO stage					
Stage I–II	37	8.27 ± 0.95	15.780/0.001	0.92 ± 0.11	13.140/0.001
Stage III–IV	13	16.25 ± 2.67		1.62 ± 0.27	
Lymph node metastasis					
Yes	24	17.98 ± 2.35	18.300/0.001	1.72 ± 0.28	16.250/0.001
No	26	9.54 ± 0.12		0.79 ± 0.08	

### Correlation of MATN1-AS1 and miR-200b in cervical cancer tissue

**Figure 1:**
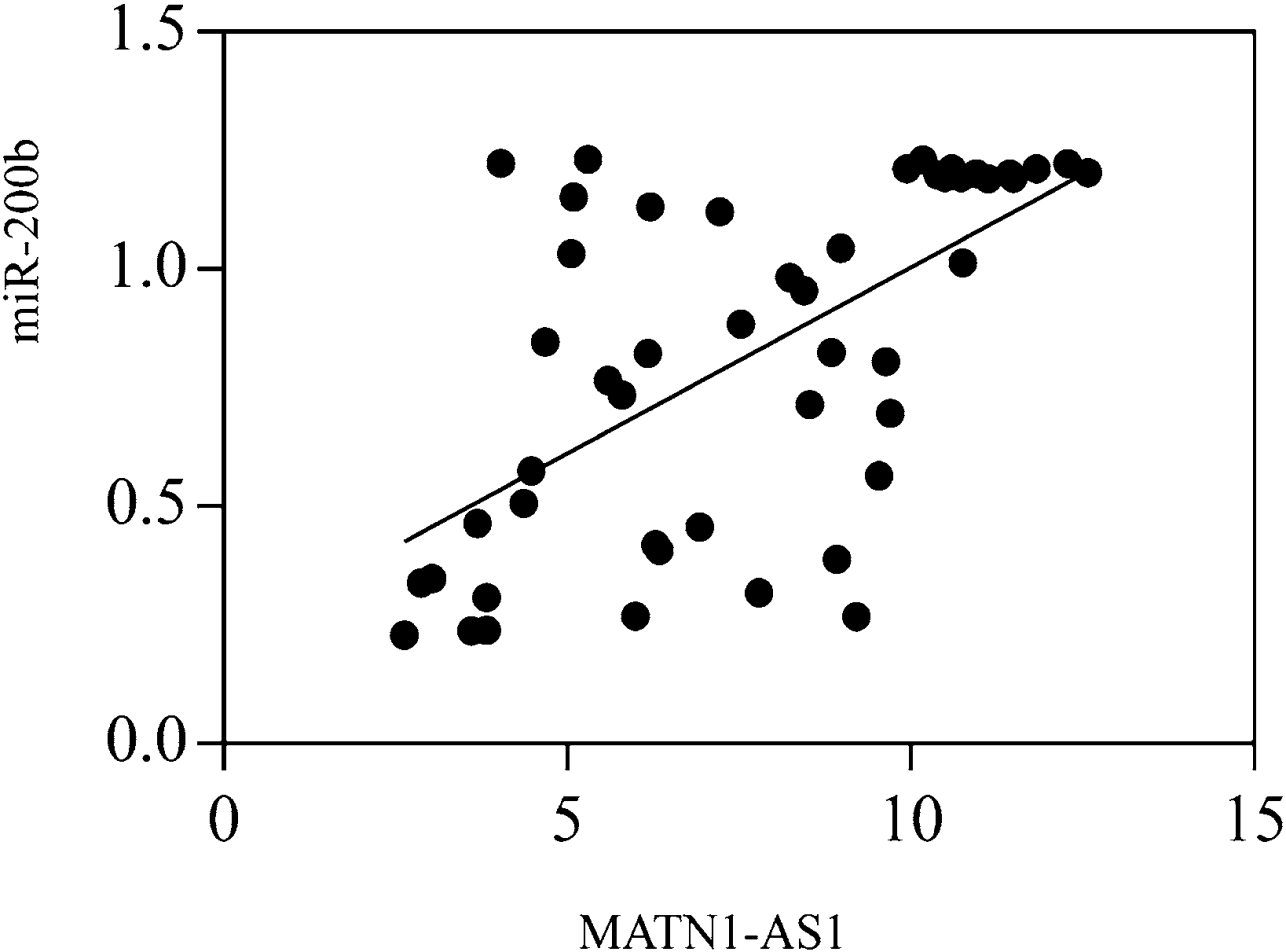
The correlation between MATN1-AS1 and miR-200b.

The correlation analysis shown that MATN1-AS1 was positively correlated with miR-200b (r = 0.625, *P* = 0.001) (Fig. 1).

### Relative expression of MATN1-AS1 and miR-200b in silenced MATN1-AS1 cervical cancer cells

Compared with the blank control group, the relative expression of MATN1-AS1 and miR-200b in the silent MATN1-AS1 group significantly decreased (*P* < 0.05) (Table 4).

**Table 4:** Relative expression of MATN1-AS1 and miR-200b in cervical cancer cells silenced with MATN1-AS1 ($\overline{x} \pm s$).

Group	Case	MATN1-AS1	miR-200b
Blank control group	10	13.05 ± 2.47	1.24 ± 0.25
Silent MATN1-AS1 group	10	7.25 ± 0.83	0.42 ± 0.05
*t*		7.039	10.170
*P*		0.001	0.001

### 3-year survival rate of cervical cancer

**Figure 2:**
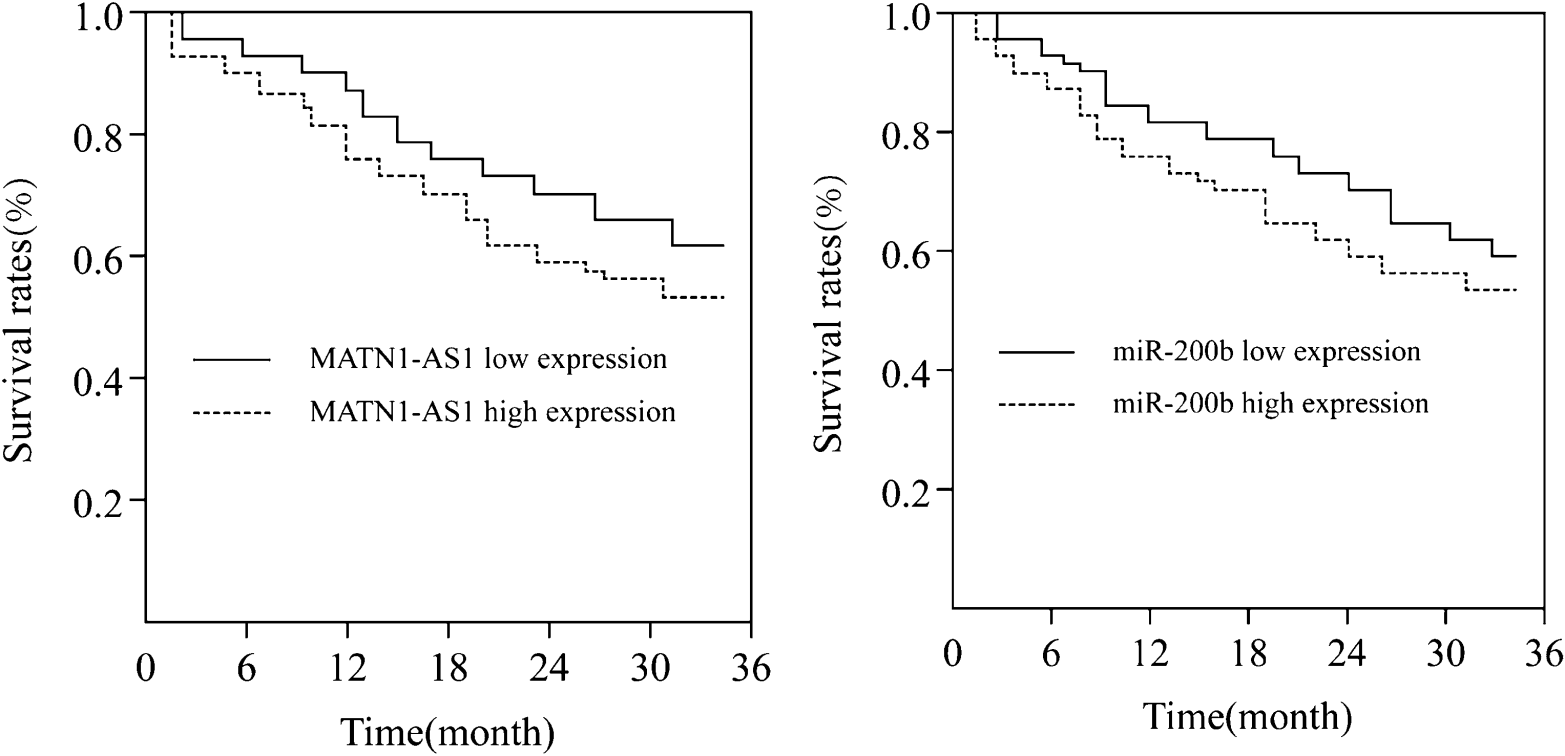
Survival curve.

The 3-year survival rate of 48 patients with cervical cancer was 66.67% (32/48) and 2 patients were lost. The survival rate of patients with high expression of MATN1-AS1 was lower than that of patients with low expression of MATN1-AS1, and the survival rate of patients with high expression of miR-200b was lower than that of patients with low expression of miR-200b (*x*^2^ = 4.251, 5.244, *P* = 0.011, 0.008) (Fig. 2).

## Discussion

Cervical cancer is one of the malignant tumors with the highest incidence rate among female tumors with high mortality rate. The clinical treatment for cervical cancer mainly includes surgery and radiotherapy. The surgery can be used for patients with early cervical cancer and radiotherapy can damage ovaries and vaginas of patients and cause a greater impact on the quality of life of young patients^8,9^. The occurrence of cervical cancer has a certain correlation with HPV infection because of HPV can invade the epithelial tissues and mucous membranes of patients and cause infection of the cervix and anus of patients. Because of the high degree of malignancy and incidence of cervical cancer that it is important to find the molecular mechanism for in-depth research on tumor development^10,11^.

Researchs shown that abnormal expression of many kinds of IncRNA plays an important role in the occurrence and development of cervical cancer^12,13^. IncRNA can regulate gene expression at different levels and participate in a variety of physiological processes such as alternative splicing, nuclear introduction, epigenetics. A variety of studies have shown that IncRNA was an important component in the carcinogenesis of cervical cancer which could affect the migration, proliferation and invasion of cervical cancer cells. MATN1-AS1 is a newly discovered IncRNA which has decreased expression in patients with ischemic stroke.

In this study, we first investigated the expression pattern of MATN1-AS in cervical cancer. We discovered that the expression of MATN1-AS is notably up-regulated in cervical cancer tissues compared with adjacent tissues, and also significantly up-regulated in cervical cancer cell lines compared with normal cervical epithelial cell line. Furthermore, we collected 50 cervical cancer patients' serums before surgery and 50 patients with benign uterine lesions were selected as the control group. We discovered that serum MATN1-AS is remarkably up-regulated in cervical cancer patients compared with healthy controls. Thus, this study provided a novel biomarker for the diagnosis of cervical cancer. We found that the expression of MATN1-AS1 in tissues and serum of patients with cervical cancer increased in the study that could lead to poor prognosis. Silencing the expression of MATN1-AS1 could reduce the expression of MATN1-AS1 and inhibit the development of tumor. Relevant research results shown that MATN1-AS1 could regulate the pluripotency, cell differentiation, apoptosis of cell stem cells, and affect epithelial mesenchymal transformation, cell invasion and metastasis, growth, and played an important role in the development of tumors^14,15^. The above results are consistent with the results of this study and it is indicating that MATN1-AS1 is a promotion sensitivity gene. the expression of MATN1-AS1 silence can inhibit the proliferation of cells and inhibit the growth of tumors.

miRNA is an endogenous RNA and relatively conservative in evolution and can regulate post transcriptional genes^16^. Research shown that the formation of miRNA related proteins interferes and participates in the invasion and migration of cells and the abnormal expression level of miRNA related proteins can lead to the occurrence of many diseases^17^. Many miRNA genes are located in tumor related gene regions and different RNAs have different roles in the occurrence, metastasis and invasion of tumors. The study found that miR-200b is a cancer promoting factor with highly expressed in cervical cancer patients. It could promote the proliferation and migration of tumor cells, and inhibit the expression of MATN1-AS1 in cervical cancer cells which could reduce the expression of miR-200b. Survival analysis shown that miR-200b was negatively related to the prognosis of patients. Relevant researchers have shown that the abnormal expression of miR-200b in tumor tissue can inhibit or promote the occurrence and development of tumors and miR-200b can regulate epithelial mesenchymal transformation and could target FoxG1 to promote the development of cervical cancer^18^. The above results are consistent with the results of this study and it is indicating that miR-200b is an oncogene that can promote the development of cervical cancer.

Research shown that miRNA and IncRNA could form a ceRNA regulatory network and IncRNA can competitively bind to miRNA through miRNA reaction elements to regulate miRNA and downstream genes^19^. Studies have shown that the IncRNA in the cytoplasm can bind to miRNA as ceRNA which blocks the regulation of miRNA on target mRNA and the miRNA in the cell can interact with the IncRNA to affect the development of tumors^20,21^. we found that MATN1-AS1 and miR-200b have a potential role relationship in the study. MATN1-AS could promote the proliferation of cervical cancer cells, inhibit cervical cancer cells apoptosis, inhibit the expression of MATN1-AS in cervical cancer cells, and reduce the expression of miR-200b indicating that MATN1-AS1 could promote the occurrence and development of cervical cancer as miR-200b ceRNA and they were related to the prognosis of patients.

To sum up, MATN1-AS1 and miR-200b are highly expressed in cervical cancer and are positively correlated which can affect the prognosis of patients. We uncovered that MATN1‐AS1 as a ceRNA to regulate miR‐200b in cervical cancer for the first time.MATN1-AS1 can be used as the ceRNA of miR-200b to promote the occurrence and development of cervical cancer and is a new target for clinical treatment of cervical cancer. However, because the sample size is too small that have not been analyzed its mechanism of action, it is necessary to further analyze to MATN1-AS1 for involvement in cervical cancer as the ceRNA of miR-200b of the specific mechanism of development will benefit more patients with cervical cancer.

## Data Availability

All data generated or analysed during this study are included in this published article.

## References

[1] Honglan C, Yi W, Tao H, Yuming W (2022). Potential value of G6PD activity detection in the peripheral blood for the diagnosis and prognosis of cervical cancer patients with high risk human papillomavirus infection. J. Kunming Med. Univ=..

[2] Chunqing W, Junhua M, Zhi'an Z, Li W, Lijun S, Hongbing C (2022). Combined detection value of serum Bmi-1, SCC Ag and HPV-DNA types for the diagnosis of cervical cancer. J. Clin. Exp. Med..

[3] Qingjing W, Changzhao C, Bo L, Liwei L, Xueping Y (2022). Study on the diagnostic efficacy of transvaginal color doppler ultrasound and three-dimensional color power angiography in the staging of cervical cancer. Chin. J. Ultrasound Med..

[4] Chenyang Z, Qiong L (2019). Effect of lncRNA MAFG-AS1 and miR-143-3p on the proliferation and apoptosis of cervical cancer cells and its mechanism. Chin. J. Oncol. Prev. Treatm..

[5] Gollavilli PN, Parma B, Siddiqui A, Yang H, Ramesh V, Napoli F (2021). The role of miR-200b/c in balancing EMT and proliferation revealed by an activity reporter. Oncogene.

[6] Yinbing T, Heteng Z, Wenbo Z, Pengcheng J (2022). Research progress on roles of lncRNA as ceRNA in EMT of gastric cancer. Cancer Res. Prev. Treatm..

[7] Vaccine and Immunization Branch CPMA (2019). Expert consensus on immunological prevention of human papillomavirus? Related diseases. Chin. J. Prev. Med..

[8] Lijuan H, Wenwen W, Jianjun Z, Yan S (2021). Serum HIF-1 α, the role of DcR3 and TSGF in the diagnosis of cervical cancer and their relationship with clinicopathological parameters. Shandong Med. J..

[9] Simeng Q, Shanzi Q, Huaping C, Zuojian H, Shan L (2021). Diagnostic value of fibrinogen, platelet distribution width, neutrophil to lymphocyte ratio in cervical cancer. Int. J. Lab. Med..

[10] Yirui C, Chaoyang G, Xinyang L (2022). Value of serum SCC-Ag, sE-cadherin and β-HCG in the diagnosis of cervical cancer. Pract. J. Cancer..

[11] Huimin Z, Xinhua Y, Hui L, Qi Z, Di B (2022). The clinical value of transvaginal SWE technique combined with colposcopy in diagnosis of cervical cancer. Hebei Med. J..

[12] Mi L, Junhui L, Yuan Y (2020). Expression and significance of miR-141 and IncRNA-H19 in cervical cancer. J. Modern Oncol..

[13] Yan Z, Juan L, Lirong Q, Hongmin H (2020). Identification of prognosis related long strand non coding RNA in patients with cervical cancer by bioinformatics analysis. Chin. J. Clin. Exp. Pathol..

[14] Shengping L, Liming H, Feng M (2021). IncRNA HOTAIR regulates adrenal tumor cell proliferation and apoptosis through the JAK2/STAT3 pathway. J. Clin. Exp. Med..

[15] Xiaomin Z, Ying X, Shaoxiu M (2021). Expression of IncRNA-UCA1 and lncRNA-GAS5 in ovarian cancer tissues and its relationship with clinicopathological characteristics and prognosis. J. Clin. Exp. Med..

[16] Zheng W, Hou G, Li Y (2021). Circ_0116061 regulated the proliferation, apoptosis, and inflammation of osteoarthritis chondrocytes through regulating the miR-200b-3p/SMURF2 axis. J. Orthop. Surg. Res..

[17] Zhang F, Cheng N, Du J, Zhang H, Zhang C (2021). MicroRNA-200b-3p promotes endothelial cell apoptosis by targeting HDAC4 in atherosclerosis. BMC Cardiovasc. Disord..

[18] Li Y, Wei S, Zhang Z (2021). MicroRNA-200b relieves LPS-induced inflammatory injury by targeting FUT4 in knee articular chondrocytes in vitro. Exp. Ther. Med..

[19] Chunli D, Kai L, Wei S (2021). Significance of expression of IncRNA HOTAIR in serum of patients with esophageal squamous cell carcinoma. World Chin. J. Digestol..

[20] Guolei C, Haiwen N, Lili H, Ronghui M, Turxunjiang LT (2022). The mechanism of IncRNA-miR210HG in the occurrence of NSCLC. J. Modern Oncol..

[21] Zi L, Wang Y, Wen X, Li C (2022). IncRNA GK-IT1 influences the carcinogenesis of non-small cell lung cancer cells through regulating aldolase A. J. Shanghai Jiaotong Univ..

